# Arsenic speciation by using emerging sample preparation techniques: a review

**DOI:** 10.55730/1300-0527.3590

**Published:** 2023-06-23

**Authors:** Muhammad Saqaf JAGIRANI, Mustafa SOYLAK

**Affiliations:** 1 Department of Chemistry, Faculty of Sciences, Erciyes University, Kayseri, Turkiye; 2 Institute of Green Chemistry and Chemical Technology, School of Chemistry and Chemical Engineering, Jiangsu University, Zhenjiang, P. R. China; 3 School of Materials Science and Engineering, Jiangsu University, Zhenjiang, P. R. China; 4 National Center of Excellence in Analytical Chemistry University of Sindh, Kayseri, Turkiye; 5 Technology Research and Application Center (ERUTAUM), Erciyes University, Kayseri, Turkiye; 6 Turkish Academy of Sciences (TÜBA), Ankara, Turkiye

**Keywords:** Arsenic, sample preparation, preconcentration, solid-phase microextraction, liquid-phase microextraction

## Abstract

Arsenic is a hazardous element that causes environmental pollution. Due to its toxicological effects, it is crucial to quantify and minimize the hazardous impact on the ecology. Despite the significant advances in analytical techniques, sample preparation is still crucial for determining target analytes in complex matrices. Several factors affect the direct analysis, such as trace-level analysis, advanced regulatory requirements, complexity of sample matrices, and incompatible with analytical instrumentation. Along with the development in the sample preparation process, microextraction methods play an essential role in the sample preparation process. Microextraction techniques (METs) are the newest green approach that replaces traditional sample preparation and preconcentration methods. METs have minimized the limitation of conventional sample preparation methods while keeping all their benefits. METs improve extraction efficacy, are fast, automated, use less amount of solvents, and are suitable for the environment. Microextraction techniques with less solvent consumption, such as solid phase microextraction (SPME) solvent-free methods, and liquid phase microextraction (LPME), are widely used in modern analytical procedures. SPME development focuses on synthesizing new sorbents and applying online sample preparation, whereas LPME research investigates the utilization of new solvents.

## 1. Introduction

Arsenic is the most hazardous element spread as a result of natural occurrences and anthropogenic actions. It has received a lot of attention worldwide due to its toxicological effect. In aquatic environments, its presence can be attributed to various sources such as surface weathering, underground deposits of arsenic-rich geological sources, biological activities, volcanic emissions, and anthropogenic activities [1]. Arsenic compounds are used in various industries, such as herbicide/pesticide production, mining operations, semiconductor manufacturing, and wood preservation. Arsenic (As) concentration in the environment has risen above a WHO-permissible level, which causes long-term mutilation, and about 250 million people worldwide have been affected by its toxicity [2,3]. As exposure can result in significant health problems such as cardiovascular illnesses, neurological disorders, hematological impacts, and lung, bladder, kidney, and liver malignancies. In 2001, the USEPA and the WHO decreased the permissible limit of As in fresh water from 50 ug/L to 10 ug/L [4]. To minimize the toxic effects of As on humans, it is necessary to monitor the quantity of As in the environment [5]. Several analytical methods have been reported for the elimination and quantification of As, such as GF-AAS [6], AFS [7], and ICP-OES [8]. ICP-MS [9], GC [10], HPLC [11,12], and CE [13], coprecipitation [14], and solvent extraction techniques (liquid-liquid extraction and dispersive liquid-solid phase sorbent-based extraction). Despite the significant development in analytical methods, sample preparation is still crucial for determining target analytes in complex matrices [15–17].

**Figure 1:**
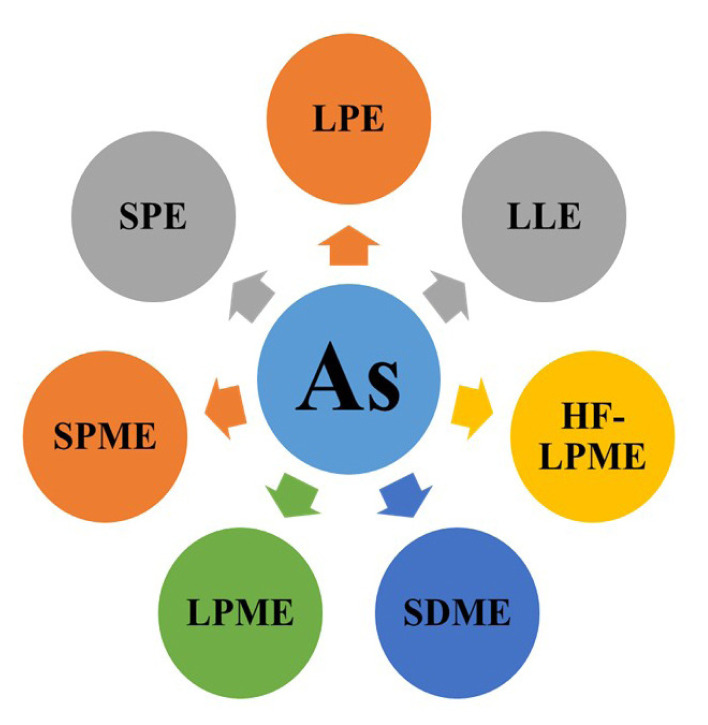
Different methods used for the extraction of As.

The abovementioned sample pretreatment techniques have some drawbacks, such as being time-consuming and expensive and using a larger amount of volatile solvents. Researchers focus on developing new, economically beneficial, easy-to-operate, and fast operation processes and environmentally friendly methods that fulfil the rules of Green Analytical Chemistry (GAC). GAC has become more important and significantly impacted all analytical methods. GAC principles focus on reducing energy use, miniaturizing the methods to be simpler and more automated, using less toxic reagents, integrating analytical methods, minimizing the sample size, and not needing derivatization [18]. Analytical methodologies and techniques have been seriously promoted and developed due to the advancement in information and instrumental technologies [19]. More work is being done to improve sample preparation, and advancements in analytical chemistry are moving toward making the sample preparation stage for analysis more environmentally friendly. Several methods have been reported to prepare samples “green” [20], but one of the most important goals is to reduce the size of analytical processes and make them faster and easier. The next important thing is to use green media instead of volatile solvents, such as ionic liquids, agro solvents, and supercritical fluids. Different factors affect the sample preparation process, such as microwave and UV radiation, high temperature and pressure, and ultrasound energy. It is essential to optimize these parameters to obtain excellent extraction efficiency. Therefore, it is important to develop a way to prepare samples that keeps the effective ingredients at the highest level and separates and eliminates the ineffective ingredients and impurities [19,20]. Different modern extraction methods have been widely used for sample preparation. For example, solid phase microextraction (SPME) and liquid phase microextraction (LPME) fulfil the requirements of researchers and also are in line with the rules of GAC; they are a miniaturized form of SPE and LPE techniques; they are fast, easy, environmentally friendly, and economically beneficial. These have the highest score in comparing analytical procedures because they are more environmentally friendly and cost-effective [21–24]. In addition, microextraction methods have higher selectivity and sensitivity, with excellent extraction performance and enrichment factors. Figure 1 represents arsenic extraction methods.

## 2. Microextraction techniques

Sample preparation is crucial in sample analysis, but it takes too much time and requires extra protocols. The traditional methods for preparing samples are not in line with the goals of GAC because they use a lot of volatile solvents, produce dangerous wastes, and harm the environment [25]. Microextraction techniques (METs) are the newest green approach that replaces traditional sample preparation and preconcentration methods [26,27]. METs have minimized the limitation of conventional sample preparation methods while keeping all their benefits. METs are the miniaturized form of basic extraction methods, like SPE and LLE [28]. Even though METs can be very different, they all have something in common. For example, they improve extraction efficacy, are fast, automated, use less amount of solvents, and are suitable for the environment [29].

One of the most important things that affect how well METs work is the choice of the proper extraction phase. At the moment, METs use several adsorbents and solvents as extraction media [30]. One of the critical steps in microextraction is sample preparation and analysis. Quantitative analyses often rely heavily on the precision and accuracy of sample preparation. The pretreatment performance aims to eliminate interference from potential species and boost the final signal of the analyte through amplification. Preconcentration of the targeted analyte through extraction from a complex matrix into a small volume of extracting solvents can improve the analytical signal. Through the use of a suitable sample preparation method, it will be possible to analyze more complex samples and minute quantities of the objective analyte. Analyte transfer from the first solution into a second phase is the basis of all sample preparation techniques. The second phase, the acceptor phase or the extracting phase, must be compatible with the final analysis instrument (for instance, an aqueous acceptor phase cannot be injected into a gas chromatograph). In addition, the analyte needs to be soluble enough in the extracting phase, and picking an extracting phase with a low enough volume can cause preconcentration of the analyte [31]. Minimal amounts of solid adsorbent are used in SPME for analyte extraction.

**Figure 2:**
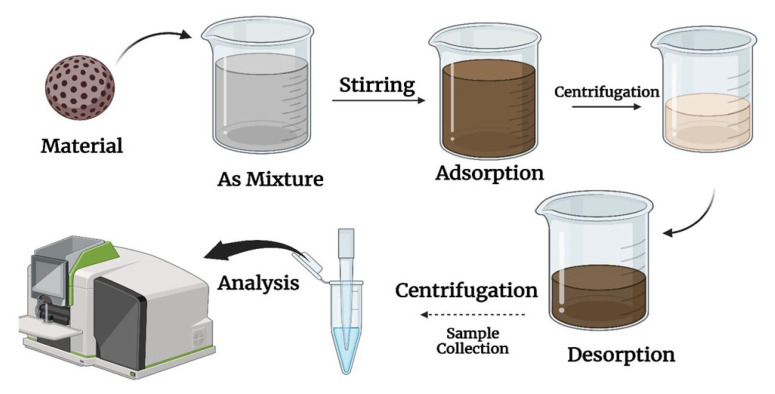
Schematic representation of microextraction of As.

A few microliters of a solvent could be used to desorb the sorbent before GC analysis, or the sorbent could be introduced directly. This method is fast compared to the SPE, in contrast to SPME. This technique simplifies the sample preparation process and minimizes interferences during the complex sample analysis. METs can be generally categorized into two groups: LPME and SPME. Pawliszyn et al. initially presented the SPME method in 1990 [32]. This method separates the analyte into a sample and an extracting phase on a fused silica fiber [33]. Figure 2 shows the general schematic diagram of the microextraction of As.

### 2.1. LPME

LPME has been widely used for the sample preparation process due to its quick phase transportation, high extracting efficiency, ease of modification, and the possibility of direct injection of the final phase for analysis; it is the cheaper, highly stable extracting phase, and has memory effects. The LPME method can be hyphenated with modern analytical techniques such as GC, HPLC, and CE [19]. While there are many other important techniques, the most common LPME methods are SDME, DLLME, and HF-LPME. Several methods have been proposed to stabilize the extracting phase, prepare separated phases, and improve extraction efficacy in LPME; this is only possible on a microliter scale. The extracting phase in two-phase LPME comes into direct contact with the sample solution, which can speed up the extraction process but also lowers selectivity, necessitates more extensive sample clean-up, and restricts the extracting solvent to water-immiscible organic liquids. In contrast, a third solvent was used in three-phase LPME that is immiscible with both the sample solution and the final acceptor phase. This arrangement significantly improves the method’s selectivity and enables using the aqueous acceptor phase. In low-pressure, in mixed-element LPME reactors, the liquid extraction phase is stabilized. Additionally, some LPME methods were developed to expand the contact area between the phases and boost the extraction driving force, both of which contribute to the overall speed and efficiency of the process [34]. The LPME technique preserves the analyte’s relatively high EF while combining sampling, isolation, extraction, and concentration in a single step. LPME has been utilized for several different things, including the extraction of analytes from water samples, including organic substances such as pesticides [35], polycyclic aromatic hydrocarbons (PAH) [36], personal care products and pharmaceuticals [37], and sulfur-based compounds [38], toxic metal ions like As^3+^ and As^5+^ [39, 40], vanadium [41], and fluorine [42]. The LPME technique is frequently used with samples with complex matrixes identified by their diverse, low-level component contents. These samples include natural products [43], food [44], and biological samples [45]. They highlight the potential of LPME techniques as a potential sample preparation tool in complex sample analysis. Different types of LPMEs, such as SDME, HF-LPME, and DLLME, are the three main categories to increase the stability and dependability of LPME; the extraction phase is housed inside a porous hollow polypropylene fiber using this method [46]. The foundation of DLLME is the application of a dispersive extraction solvent. When the water sample is mixed with the extraction solvent, droplets of the extractant can form. This method expedites the movement of analyte from the sample to the extraction stage [47]. All METs rely on transporting sample-bound analytes into an acceptor-phase reaction for extraction on liquid or solid phase. All these methods work well and fulfill the needs of modern eco-friendly analytical procedures [48].

#### 2.1.1. Single-drop microextraction

SDME was proposed to address the issue of the increasing size of the extraction solvent [49]. Analytes in the sample solution are thought to be partitioned into the extractant solvent droplet dangling from the syringe needle tip, making SDME possible. The extracting phase can contact the sample solution or headspace [50]. Traditional SDME was an innovative LLE-based technique, but newer setups have been created to make the process easier and less cumbersome. In response to this problem, Zhang et al. [51] proposed ultrasonic nebulized headspace solid-phase microextraction (HS-SDME). They demonstrated that the analyte’s gas-liquid distribution equilibrium is rapidly attained and enhanced by forming an “ultrasonic fountain” through ultrasonic vibration through solvent extraction.

Zhang et al. [52] presented a dynamic format similar to linking the evaporated species to the extracting droplet. As a sample pretreatment, extraction, and preconcentration method, SDME has achieved near-universal acceptance. However, due to the two-phase extraction mechanism, its direct immersion mode is less selective and has less clean-up ability. In addition, nonvolatile compounds cannot be extracted using the headspace mode. By suspending the extracting droplet in an organic liquid membrane, Cantwell first introduced the three-phase format of SDME using an optical probe as the microdrop holder; online monitoring of the target analyte by UV/Vis detection was also presented following SDME procedure [53].

#### 2.1.2. Dispersive liquid-liquid microextraction

The DLLME technique is implemented by inducing the formation of tiny droplets of an organic solvent that acts as an extracting agent within the sample. This is achieved by utilizing a disperser solvent that exhibits complete miscibility in both the aqueous and organic phases. The extensive contact surface area between the model and the extracting degree facilitates the efficient extraction of the target analytes. Novel advancements in the DLLME configuration have been introduced to streamline and expedite the extraction procedure [54]. Ebra Himpour et al. [55] proposed a novel approach to address the time-consuming centrifugation step in the DLLME method, which involves the separation of the dispersed solvent for phase separation. The proposed process uses an in-line filter to separate the organic extracting phase from the aqueous sample.

Furthermore, the utilization of hollow fiber was employed for phase collection extraction. The elution of fiber was carried out using acetonitrile, and the resulting solution was subsequently introduced into the analysis instrument [56]. Lopes et al. [57] employed a combined hollow fiber-dispersive liquid-liquid microextraction (DLLME) technique to extract and collect analytes simultaneously. This involved inserting a hollow fiber with a porous wall impregnated with 1-octanol into the sample containing the extractor and disperser solvent mixture at the onset of the extraction process. The analytes were extracted from the membrane through liquid desorption, which was carried out by introducing acetonitrile.

#### 2.1.3. Hollow fiber liquid-phase microextraction

The initial application of hollow fiber-based liquid-phase microextraction (HF-LPME) was aimed at facilitating a liquid-liquid microextraction process involving three phases. Hollow fiber presents two distinct partitions, the wall pores and the lumen, which can accommodate a single extracting solvent or two nonmixing solvents for two-phase or three-phase extractions, respectively [58]. The extraction solvent is stabilized, and leakage is prevented during the process due to the porous nature of the hollow fiber. Therefore, this methodology has the potential to be replicated and yield greater precision when contrasted with SDME. The initial three-phase liquid-liquid microextraction (HF-LPME) was documented to have employed an organic solvent and an aqueous solution. Alternatively, using two immiscible organic solvents, the three-phase approach in HF-LPME is feasible [59]. The HF-LPME method, compatible with gas chromatography, utilized n-dodecane and either acetonitrile or methanol as solvents in the wall pores and lumen, respectively. The extracted phase was drained from the lumen and injected into the analysis instrument. Dynamic HF-LPME can enhance analyte mass transfer and accelerate extraction. Various configurations were introduced to facilitate the dynamic movement of the sample solution or acceptor phase [60]. The dynamic device developed by Pimparu involved the sample solution’s initial passage through the hollow fiber’s lumen, thereby facilitating the diffusion of the analyte into the immobilized organic solvent. Subsequently, the lumen was extracted using the final solution, and the resulting solution was introduced into the analytical apparatus [60]. HF-LPME is a viable microextraction methodology for examining intricate matrices or when the sample solution is available in a restricted volume. Despite the advancements made in HF-LPME, this technique continues to be hindered by its reliance on a passive diffusion-based extraction mechanism that operates relatively slowly.

Table 1 represents the application of LPME for arsenic species [61–81].

### 2.2. SPME

SPME is an emerging extraction technique introduced in 1990 [82]. SPME has been recognized as a modern alternative to conventional sample preparation techniques and exhibits various potential applications [83–85]. The SPME methodology entails the adsorption of the desired analytes onto the exterior of a fiber that has been coated. After the extraction process, the analytes that have been trapped are subjected to desorption in order to facilitate their analytical determination. The desorption process is commonly executed through a thermal technique, whereby the SPME fiber is inserted into the injection port of the GC. The SPME method combines with LC or CE. The SPME coating material can maintain its structural integrity during desorption, enabling autonomous operation and repeated utilization. For instance, an appropriate sorbent must be chosen to successfully apply the aforementioned SPME procedures in various settings. Other essential factors have been optimized in the SPME process for the selective extraction, reusability, and disposal of the sorbents with multiple analytes in environmental samples [86]. Due to its benefits, SPME has emerged as a viable alternative for detecting and specifying heavy metals. Fiber-based SPME [87], in-tube SPME [88], in-tip SPME [89], stir sorptive bar extraction [90], and dispersive SPME [91] are the five most common ways to run SPME. Over the past decade, several papers are detailing the utilization of SPME in environmental analysis. The comprehensive establishment of the SPME technique needs several intermediary procedures spanning from sample acquisition to conclusive outcomes, encompassing sample pretreatment, SPME, desorption, and detection. The objective of this particular stage is to purify and enhance the sample, thereby achieving high selectivity and sensitivity. Several SPME configurations have been devised to achieve this objective, including in-tube, fiber, in-needle, in-tip, thin-film, and stir-bar microextraction. The selection of configurations can be tailored to a particular objective, considering their respective benefits and drawbacks. One of the benefits of SPME is its solvent-free nature, making it a more environmentally friendly option [23].

Additionally, SPME is an online method, allowing for real-time analysis. Furthermore, this technique is particularly well-suited for the study of volatile compounds. The first method relies on directly immersing the adsorptive materials into the samples. In contrast, the second method relies on the adsorptive extraction of volatile or semivolatile molecules from the headspace of the samples. In both instances, the utilization of adsorbent materials has served as a driving force behind the researchers’ efforts to enhance the effectiveness and selectivity of the SPME procedures.

#### 2.2.1. Direct immersion method (DI-SPME)

The DI-SPME technique involves the direct interaction of the adsorbent materials with the sample matrix that offers the desired analytes. It is imperative for adsorbent materials to effectively isolate all target analytes in a given sample while eliminating any interfering compounds from the matrix. Regarding this matter, the adsorbent materials must possess adequate sorption capacity to adsorb all the intended analytes and exhibit targeted interaction. Subsequently, the complete release of the target analytes can be achieved through elution by an organic solvent or an increase in temperature before their introduction into the detection system. Moreover, implementing new adsorbent materials, such as nanomaterials (NMs) with elevated surface areas, may represent an optimal approach for enhancing sorption capacity. Nanosorbents are widely utilized in environmental contexts due to their characteristics, including high sorption capacity and, in certain instances, high selectivity [92].

#### 2.2.2. Headspace method (HS-SPME)

The experimental procedure involves the placement of the sample into a vial that is subsequently sealed with a cap. The target volatile or semivolatile analytes are extracted by exposing the adsorbent materials to the sample’s headspace, which may be in a gas, solid, or liquid state. This approach enhances selectivity by mitigating the impact of the high number of volatile substances in environmental samples. The optimal nanomaterials for headspace (HS-SPME) are identical to the adsorbents utilized in the direct immersion (DI-SPME) technique. The desorption process employed in HS-SPME typically involves thermal desorption, necessitating that the nanomaterials exhibit robust chemical and thermal stability at the relevant desorption temperatures. SPME remains the most commonly used microextraction technique [93]. The SPME device, widely found in both classical and commercial contexts, comprises a fiber of fused silica or stainless steel coated or uncoated with a thin sorbent layer. The fiber is attached to a device that resembles a syringe. Researchers have employed various techniques to enhance the efficiency of SPME in environmental contexts by utilizing numerous adsorbent materials such as polymeric materials, molecularly imprinted polymers, and nanomaterials. The utilization of NMs in SPME methods has garnered significant interest in the analytical and environmental domains owing to their improved characteristics and ability to provide high selectivity for specific analytes. The utilization of SPME by NMs for ecological sample clean-up and pretreatment is experiencing a rise in interest [94]. Table 2 shows the application of SPME for arsenic speciation [11,67,80,95–102]

## 3. Arsenic speciation using different modern sample preparation techniques

The primary origin of inorganic arsenic is attributed to rock weathering in nature, while anthropogenic activities also contribute to its release. The occurrence of inorganic arsenic in groundwater is predominantly in the nonionic trivalent (As(III)) and ionic pentavalent (As(V)) forms, with varying proportions depending on the environmental conditions of the aquifer [103]. Determining arsenic speciation in water is commonly regulated by factors such as redox conditions, pH, biological activity, and adsorption reactions [104–106]. Under low Eh conditions, arsenic is transformed into its As(III) form, whereas under high Eh conditions, As(V) is the predominant arsenic species. According to research, As(III) is more hazardous than As(V) and poses a challenge in terms of its elimination from water using conventional methods [107,108]. Typically, the elimination of As(III) involves an initial oxidation to As(V), followed by the removal of As(V) through adsorption, precipitation, or ion exchange techniques [10]. The environment is exposed to organic arsenic species, specifically monomethyl arsenic acid (MMA) and dimethylarsinic acid (DMA), primarily as a result of agricultural and industrial practices [11]. MMA and DMA are active constituents in weed control and defoliation agents frequently employed before cotton harvesting. These constituents have been identified as problematic pollutants in groundwater at locations where pesticide manufacturing and inappropriate disposal practices have been carried out, as per previous research [12]. The methylation process of inorganic arsenic has been suggested as a potential biological mechanism for detoxification. However, recent studies have revealed that methylated arsenic species can induce DNA damage, chromosomal aberrations, and tumor promotion in rodents [13,14]. Although natural waters are primarily composed of inorganic species, the occurrence of MMA and DMA has also been documented in the literature [12,15].

Arsenic toxicity is primarily influenced by its chemical composition and degree of oxidation. Arsenite As^3+^ and arsenate As^5+^ are regarded as toxic and carcinogenic forms. Arsenocholine (AsC) and arsenobetaine (AsB) are regarded as nontoxic arsenic compounds. In contrast, monomethyl arsenic acid (MMA) and dimethylarsinic acid (DMA) are less toxic than inorganic arsenic species As^3+^, As^5+^, MMA, DMA, AsC, and AsB. [109]. As^3+^ accumulates quickly in biological systems. Both oxidation states prevent the mitochondria from performing tasks that require energy. As^3+^ substances have a strong affinity for the sulfhydryl groups of proteins and render enzymes inactive. As^5+^ competes with phosphate in cellular reactions and can decouple oxidative phosphorylation, causing the high-energy bonds of adenosine triphosphate to be broken. As can generally lead to both acute and chronic poisoning. According to recent studies, trivalent methylated arsenic species are more toxic than their inorganic counterparts. The quantification of total As cannot aid as a reliable data source on As toxicity. Total As could be determined until 1973, but since then, As3+ and As5+ species have been determined using the hydride formation technique [110]. Altunay et al. [111] reported that VA-LPME is based on the DES (DES-VA-LPME) for the sample preparation and preconcentration of As from honey water and rice. AAS-EDDS have been used for the quantification of As from the prepared sample. Different parameters like pH, DES type, chelating agent amount, emulsifier agent’s quantity, molar ratio and volume, sample volume, and vortex time have been optimized to attain the maximum efficiency of the proposed sample. The DES-VA-LPME method has obtained a LOD of 6.5 ng/L, a quantification limit of 20 ng/L, and an EF of 70. The proposed method has been successfully used to extract As from real samples. Jiang et al. [64] proposed an HF-LPME method using ammonium pyrrolidine dithiocarbamate (APDC) as an extractant material for eliminating As^3+^ and As^5+^. For the quantification of As^3+^ and As^5+^, ETAAS has been used. Different factors have been optimized to obtain maximum extraction efficiencies, such as pH, selection of organic solvent, the volume of APDC, stirring rate, and time study. At the optimized conditions, the detection limit was up to 0.12 ng/mL with an EF 78, and the LOD value was 5 ng/mL. The developed method has been used to determine the As^3+^ and As^5+^ in hair, fresh water, and spiked samples; the percentage recovery was obtained up to 86%–109%. Chamsaze et al. [61] developed HS-SPME to extract As from water samples. GF-AAS has been used for the quantification of As. The typical concentration was 30 pg/mL, equal to a typical mass of 0.12 pg/mL, and the calibration curve was linear from 45 pg/mL to 4 ng/mL. The testing results for As in tap water, reference materials, and washing machine powder showed that the method presented was accurate, obtained good results, and was applied in real application. Haghnazari et al. [112] proposed a counter-current liquid-liquid microextraction (CLLME) based new method for eliminating As from biological samples such as plasma and urine. ETAAS have been used for the quantification of As. The LODs) were found up to 0.03–0.05 g/L and linear in the 0.1–50 g/L range; EF ranged from 198 to 212 and 220 to 240, respectively. Based on seven replicate measurements of 5.0 g/L of As, the method’s repeatability (intraday) and reproducibility (interday) fell between 2.3% and 3.5% and 4.0% and 5.7%, respectively. The developed method for speciation of As from plasma and urine was found to have excellent capability, as evidenced by the percentage recovery obtained up to the 92%–107% range. Majumder et al. [113] reported an HF-LPME-based method for extracting As from water samples. TXRF spectrometry has been used for the analysis of As. Relevant experimental parameters have been evaluated during As extraction, such as pH, extractant type, solvent type, and agitation time; it was observed that As^3+^ extraction was more efficient at pH 13, whereas As^5+^ extraction was best at pH 8.5. The proposed method was also used to preconcentrate and determine As from environmental samples. Zeng et al. [114] used Triton X-100-based HF-LPME to eliminate As from biological and environmental samples. FAAS has been used for the determination of As. Triton X-100 was utilized as an adsorbent. Under the optimal conditions, the LOD for As^3+^ was up to 0.08 ng/mL, the EF was up to 280, and the RSD was 5.2% for five replicate measurements of 10 ng/mL. The developed method obtained good results during the analysis of As^3+^ and As^5+^ from the biological and environmental samples. Yilmaz et al. [115] reported a novel approach merging the principles and benefits of the SPME method, hydrolytic enzymes, and ultrasonic radiation power used to develop and improve green sample preparation techniques. This new method is called hydrolytic water phase microextraction assisted by ultrasound (UA-EH-WPME). This solvent-free method is an innovative, environmentally friendly, and straightforward sample preparation process for extracting As from food (rice and flour) samples. The prepared sample has been analyzed by the ICP-MS with LOD up to 27.3 g/kg and RSD of 4.27%, respectively. Shirkhanloo et al. [98] proposed USA-DSL-MPME method was used for the total arsenic (TOA) analysis from water and urine samples. As an extraction phase, bulky amine-functionalized bimodal mesoporous silica nanoparticles (NH_2_-UVM7) were submerged in ionic liquid (IL). Then, a syringe was used to inject the mixture of NH_2_-UVM7 and IL/acetone [HMIM][PF6]/AC into 10 mL of the sample with As^3+^, As^5+^ TOA, and a pH of 3.5. After ultrasonication and centrifugation, the ILs settled with nanoparticles of NH_2_-UVM7. The As^5+^ anion was removed from the centrifuge tube by binding to the amine group As^5+^ + NH_2_-UVM7). The total amount of As was found after oxidizing As^3+^ to As^5+^ with KMnO_4_ solution in an acidic medium. ET-AAS was used to quantify the As^5+^; after that, KOH solutions were used to extract it from the separated phase. Under the best conditions, the linear range (LR), the LOQ, RSD%, and EF for As^5+^ were 0.02–1.65 g/L, 11 ng/L, 4.3%, and 100.5, respectively. The proposed method worked well to find As^3+^ and As^5+^ in water and biological samples such as urine, with recoveries in the 95%–103% range. By using certified reference material in urine, the accuracy of the USA-DSL-MPME method was also confirmed, with recoveries in the 95%–102% range. Chen et al. [80] proposed HF-SPME online coupled with (IP-RP-HPLC- ICP-MS) developed for the As analysis. The sampling frequency was 6.5 h1, with LOD six target As species were found up to 0.017–0.053 g/L, the RSD cAs^3+^, DMA, AsB, AsC = 0.5 g/L, (cAs(V), MMA = 0.1 g/L, n = 5) ranged between 3.1% and 8.7%, and the EF varied from 4- to 19-fold. To check the analytical reproducibility of the proposed method, reference materials such as DORM-2 (dogfish) and CRM No. 18 (human urine) have been analyzed. The proposed method has been efficiently utilized for the As analysis from human urine, with recoveries ranging from 92.6% to 107% for spiked samples. Ahmad et al. [67] proposed a method for analyzing trace amounts of As using DSPME that uses ultrasound. At a concentration of 100 g/L of arsenic, the cadmium sulfide nanoparticles (CdS NPs) made by hydrothermal synthesis took in both types of arsenic within 20 s. The LOD of the proposed method (3 S/m) was found to be 0.5–0.2 ng/L for As^3+^ and 0.8–0.2 ng/L for As^5+^. Analysis of the standard reference material (SRM) (>95% recovery with 5% RSD) showed that the method was correct when it came to systematic and constant errors. Tyburska et al. [97] proposed a hydride generation HS-SPME technique developed to determine total arsenic and selenium. The LOD was found to be up to 0.1 for As and Se, were 0.8 ng/mL 0.1 and 0.8 ng/mL, respectively. The LOD was found up to 0.8 ng/mL for As and 0.1 ng/mL for Se. The developed method determined total As and Se in real-world samples. Zounr et al. [116] developed a green and highly efficient DES-UALPME for the speciation of As^3+^ and As^5+^; DDTC was used as a chelating solvent, tetrahydrofuran (THF) was used as a dispersive solvent, choline chloride-phenol was used as an extracting solvent, and ETAAS was used to analyze the concentration of As. The developed method obtained excellent recoveries of As^3+^ and As^5+^. The LOD, LOQ, PF, and RSD were calculated as 10 ng/L, 33 ng/L, 25, and 4.3%, respectively. The developed technique’s accuracy was tested using certified reference materials. Chen et al. [100] proposed chip-based magnetic-SPME to analyze As^3+^ and As^5+^. A sequential elution strategy was used to separate MMA, DMA, As^5+^, and As^3+^so that ICP-MS could be used to measure them online. The lowest amounts found for As^5+^, DMA, MMA, and As^3+^ were 4.8, 6.3, 3.8, and 7.1 ng/L. Between 23 and 25 enrichment factors were used, and 7 samples per hour were processed. The online system was used to monitor As species in SCC-7 in cell samples. Ali et al. [102] loaded the polystyrene polydimethylsiloxane into the syringe system’s micropipette tip as an adsorbent to develop SPME. Standard solutions of As^3+^ and As^5+^ were passed through the adsorbent loaded in a micropipette tip to test the adsorption behaviors. As^3+^ was subtracted from the total inorganic concentration to calculate As^3+^ concentration in water samples. Different factors influencing the determination of the As^5+^ species, such as adsorbent amount, pH, adsorption capacity, adsorption and desorption study, volume of sample, eluent type, and volume, were also thoroughly investigated. The desired method’s EF and LOD for As^5+^ were 218 and 6.9 ng/L, respectively. The RSD (n = 10, C = 0.12 g/L) was 4.1%. CRM analysis confirmed the accuracy of the desired technique (Lake Ontario Water TM-28.3 and Riverine Water NRCC-SLRS-4). The desired technique was beneficial for determining the total As^3+^ and As^5+^ contents in various natural water samples. Li et al. [101] developed an SPME system with two monolithic capillary columns to separate and preconcentrate inorganic arsenic simultaneously. ICP-MS has been used for the As quantification. The As^5+^ or As^3+^ that was kept was then eluted one at a time with diluted HNO_3_ and put into ICP-MS to be measured. In this work, all factors that affect the retention and elution of As^5+^ and As^3+^ were carefully optimized. Using the system, online SPME of a 1-mL sample solution gave a signal enhancement factor of 60 for both As^5+^ and As^3+^. The RSD was 3.8% for As^5+^ and 3.2% for As^3+^. LODs were 0.005 g/L for both As^5+^ and As^3+^. The method was used to analyze the types of As in drinking water and water from the environment, and the results were good. Ashouri et al. [66] developed DLLME using task-specific ionic liquids (TSILs) to extract As. The developed method used an ionic liquid with an ammonium pyrrolidine dithiocarbamate (APDC) bond for chelation with As^3+^, then KPF6 as an anion-exchange reagent to change the As^3+^ chelated TSIL into a hydrophobic ionic liquid. Using a 2/1 w/w mixture of KI and Na_2_S_2_O_3_, As^5+^ was converted to As^3+^, and the total amount of As was then determined using the ETAAS analysis. To determine As^3+^ and total As, respectively, linear dynamic ranges of 0.2–15 ng/mL and 0.2–20 ng/mL were noted under ideal conditions. The LOD and LOQ were established to be 0.01 ng/mL and 0.0.034 ng/mL, respectively. The relative standard deviation (RSD%, n = 5) for determining As^3+^ (10 ng/mL) was 3.2%. Rabieh et al. [72] have devised a new way to microextract As^3+^ and As^5+^ species. It uses ionic liquid-based DLLME coupled with EAAS. At a pH of 2, ammonium pyrrolidine dithiocarbamate binds to As^3+^. 1-butyl-3-methylimidazolium bis(trifluoromethyl sulfonyl) imide pulls the As^3+^ out of the fine droplets. As^5+^ stays in the water phase and is turned into As^3+^. The difference between total inorganic As^3+^ and As^5+^ can be used to figure out how much As^3+^ and As^5+^. The pH values, the concentration of the chelating reagent, the types and amounts of the extraction and dispersive solvent, and the time of centrifugation were all optimized. The EF of 255, the lowest amount of As^3+^ found, is 13 ng/L, and the standard deviation for six measurements of 1.0 g/L of As^3+^ is 4.9%. The method worked well to find As^3+^ and As^5+^ in spiked natural water samples, with relative recoveries between 93.3% and 102.1% for As^3+^ and 94.5% and 101.1% for As^5+^.

Najafi et al. [117] developed the VA-SSM-SFD bed method for As’s sample preparation. The supramolecular solvents comprised reverse micelles of 1-dodecanol in tetrahydrofuran (THF). These micelles were made by combining micelles of 1-dodecanol in water samples on the spot. Under the best conditions, As^5+^ formed a complex with heterophony blue, which was directly measured. After As^3+^ was turned into As^5+^ by permanganate in an acidic medium, the concentration of As^3+^ was found by taking away As^3+^ from the total amount of As. Under ideal conditions, the calibration curve for As determination showed good linearity in the range of 5.0–140.0 g/L (R^2^ = 0.9988), and the LOD was found to be 0.4 g/L; for 20 g/L, the repeatability and reproducibility were each found to be 2.47% and 3.9%. Wang et al. [118] proposed a new magnetic ionic liquid-based air-assisted liquid–liquid microextraction (MIL–AALLME) method for the selective separation of trace amounts of As^3+^ and As^5+^ species in different aqueous samples, soil, and sediment samples before GFAAS analysis. In the MIL–AALLME method, the things that mattered, like pH, amounts of chelating agent, the effect of salt, and types and amounts of MIL, were measured and changed in a planned way. Under the best conditions for extraction, the MIL–AALLME method gave a good linear dynamic range (LDR) of 0.04–10.0 g/L. The LOD and the RSD for seven measurements of 2.0 g/L of As^3+^ using this method were 0.029 g/L and 2.5%, respectively. The developed method was used to study the different forms of inorganic arsenic in aqueous samples, soil and sediment samples, with good results ranging from 93.0% to 108.5% for the spiked samples. Wang et al. [119] proposed a fully automated magnetic stirring-assisted lab-in-syringe dispersive liquid-liquid microextraction (MAS-LIS-DLLME) for the rapid and efficient separation and preconcentration of trace levels of As^3+^ species in rice samples. GFAAS have been used for the analysis of As^3+^. The LOD for As^5+^ was 0.005 g/L. For seven replicate measurements of 2.0 g/L As^5+^, the RSD was 3.7%. The developed automated analytical procedure was successfully applied for trace analysis of As species from the rice samples and certified standard reference materials.

Shirani et al. [120] developed a centrifugeless deep eutectic solvent-based magnetic nanofluid-linked air-agitated liquid microextraction (CL-DES-MNF-AALLME). This study proposed a new analytical method that is good for the environment: coupled with ETAAS. This method can simultaneously preconcentrate heavy metals like Pb, As, Cd, and Co. In CL-DES-MNF-AALLME, the extraction solvent is a new magnetic nanofluid made of magnetic multiwall carbon nanotubes based on DES. This nanofluid can be easily separated from the medium without centrifugation. For cadmium, lead, copper, and arsenic, the lowest amounts that could be found were 4.2, 3, 3.5, and 3.6 ng/L, respectively. The proposed extraction method is confirmed by quantitative recoveries with enrichment factor in the range of 635–644.5 and with an RSD of 2.5%–3.1% (n = 7). Asadollahzadeh et al. [121] developed a DSLLME method to extract As^3+^. ETAAS has been used for the quantification. First, fractional factorial design (FFD) was used in screening experiments to find the variables significantly impacting the extraction process. After that, the important variables were optimized using a central composite design and response surface methodology.

## 4. Conclusions and future trends

In the past few years, many analytical techniques have been used to preconcentrate arsenic species in environmental and biological samples before analysis. The main goal of previous review articles is to show the two main ways arsenic species can be found: chromatography (HPLC, GC) and spectroscopy (ET-AAS, OES, AFS, ICP-MS). This review mostly talks about preparing samples, including modern methods that do not use solvents like SPME, and ways to use fewer solvents like LPME. Most of the microextraction techniques that use solid adsorbents are used in SPME procedures. Thus, new trends are linked to improvements in this method, which can also be seen in how other element species are determined. When the Analytical Eco-Scale is used to evaluate SPME methods, the scores are higher than those for SPE methods. SPME does not lose points for using solvents, but SPE does, so this technique loses more points. Synthesizing new SPME adsorbents is also not green because it requires different chemicals. However, they are putting the best inventions in this field. Researchers try to minimize the pollution made in analytical labs and make them safer. This is because large-scale production of SPME fibers made electrochemically or by electrospinning can reduce the pollution produced per fiber. Regarding “green” analytical chemistry, online systems with in-tube SPME have the same benefits since only small amounts of solvents are used to desorb analytes. Online SPME systems also have other benefits, such as better accuracy due to automated processes and less need for human labor.

Microextraction methods use very minute amounts of liquids or do not use the liquid, and only a few are used regularly to prepare samples. When LPME techniques are changed, preconcentration is often used using specially designed or chosen extractants, such as ionic liquids or organic solvents with a low melting point, but there are still some fears about how they are used, especially regarding ILs. This makes people look for new solvents, like deep eutectic solvents, which are used instead of ILs in green techniques. Compared to traditional LLE, LPME techniques require less solvent and are easier to use. They also make less waste. On the Analytical Eco-Scale, microvolumes of solvents used for extracting are worth much less than even a thousand times higher volumes of solvents used in LLE.

LPME techniques also have benefits like making it safer and reducing the amount of waste that is made. When combined microextraction techniques are used, high preconcentration factors are possible, but this benefit is tied to introducing more complicated data analysis methods. Complex sample preparation procedures often lead to less accurate results because each step adds a new chance of making mistakes. Also, when SPE is used, combined analytical techniques need more solvents, producing more waste. Therefore, these methods are only helpful when the detection limits are very low. Because of this, combined microextraction techniques are still not used in many studies. Some authors’ new ideas mainly involve tweaking existing methods. As a result, the methods presented are often very similar to those used to extract other analytes. It is also important to note that most papers only look at inorganic arsenic species, such as the difference between As^3+^ and As^5+^. Arseno-organic compounds are not often used in research, and chromatographic techniques are usually needed to determine what they are. When new studies show that some of these no-organic species are toxic, there may be more interest in analyzing samples with them. Until that happens, most papers will still be used to determine which types of inorganic arsenic are present.

## Figures and Tables

**Table 1:** The application of LPMEfor arsenic species.

Extraction phase	Analyte	Matrix	Technique	EF	LOD (ng/L)	Reference
AgDDC–pyridine:benzyl alcohol	As^3+^ As^5+^	Water samples	HS-SDME–ETAAS	150	45	[61]
Pd(II) aqueous drop	As^3+^ As^5+^	Water samples	HS-SDME–ETAAS	70	100–150	[62]
Aqueous drop	As^3+^	NWRI TM-28.3 CRM	HS-SDME–GC–ICP-MS	9.85	0.2	[63]
Toluene	As^3+^ As^5+^	Fresh water Human hair	HF-LPME–ETAAS	78	0.12	[64]
CCl_4_	As^3+^	Water samples	ETAAS	115	10.50	[65]
DSPME	As^3+^ As^5+^	Water, urine, and rice	ETAAS		0.01	[66]
CdS NPs	As^3+^ As^5+^	Water and food	ICP-OES		0.0005–0.0008	[67]
G/CeO_2_	As^5+^	Water	EDXRF		0.10–0.21	[68]
[C4MIM][PF6]	As	Eye makeup	ETAAS		0.049–0.262	[69]
IL-DLLME	As^3+^ As^5+^	White and red wine	ET-AAS	46	0.005	[70]
IL-LPME	As^3+^ As^5+^	Bottle, river, rain, tap water	ET-AAS	130	0.002	[71]
IL-DLLME	As^3+^ As^5+^	Tap and river water	ET-AAS	255	0.01	[72]
DLLME	As^3+^	Water	ETV-ICP-MS	60	0.0005	[73]
SBME	As^3+^ As^5+^	Synthetic, lake and tap water	ETV-ICP-MS	220	0.0003	[74]
USE-SFODME	As^3+^ As^5+^	Sea, river, tap water	ET-AAS	152	0.004	[75]
Alkanol-based reverse micelle-SPME	As	Water	Spectrophotometry		0.4	[76]
DLLME	As,	Honey	ETAAS	110	12	[77]
DLLME.	As^3+^ As^5+^	Water	GFAAS	45	0.036	[78]
DLLME	As^3+^ As^5+^	Water	GFAAS	115	0.01	[79]
In-tube HF-SPME	As	Human urine	ICP-MS-IP-RP-HPLC	4–19	0.017–0.053	[80]
DLLME	As^3+^ As^5+^	Fruit juices	HG-AFS		10	[81]

**Table 2:** The application of SPME for arsenic species.

Extraction phase	Adsorbent	Analyte	Matrix	Technique	EF	LOD (ng/L)	Reference
DMSPE	GO-SH	As	Water	TXRF	150	0.05–0.1	[11]
Ultrasound-assisted DSPME	CdS nanoflowers	As^3+^ As^5+^	Water, Food	ICP-OES		0.8 and 0.2	[67]
In-tube HF-SPME	MPTS–AAPTS/PSP	As^3+^	Human urine	IP-RP-HPLC	19	0.5	[80]
DLLME/DMSPE	Magnetic MWCNTs	As	Cow milk, fish liver, and water	ET-AAS	398–403	0.003–0.005	[95]
UA-DMSPE	G-COOH	As^3+^ As^5+^	Water and human serum/urine	FI-HG-AAS	5.0–50	0.002–0.02	[96]
HS-SPME	CAR/PDMS	As	Baker yeasts	ICP-OES		0.1–0.8	[97]
USA-DSLMPME	(NH2-UVM7)	As^3+^ As^5+^	Water and urine	ET-AAS	100	0.02–1.65	[98]
Dμ-SPE	(FTGCNCs)	As^3+^ As^5+^	Water	ICP-MS		1.2	[99]
MSPME	Magnetic metal-organic framework composite (MFC) and mercapto-functionalized MFC nanoparticles (MFC-SH)	As^3+^ As^5+^	Water	ICP-MS	23–25	7.1	[100]
SPME	N-(β-aminoethyl)-γ-aminopropyltriethoxysilane (AEAPTES)	As^3+^ As^5+^	Water	ICP-MS	60	0.005	[101]
SPME	Polystyrene polydimethyl siloxane polymer	As^3+^	Water	ET-AAS	218	6.9	[102]

## Abbreviations

AFS: Atomic fluorescence spectrometry
CE: capillary electrophoresis
GC: capillary electrophoresis CE, capillary gas chromatography
DES: deep eutectic solvent
DLLME: dispersive liquid-liquid microextraction
EF: enrichment factor
GC: gas chromatography
GF-AAS: Graphite furnace atomic absorption spectrometry
HPLC: high-performance liquid chromatography
HPLC: high-performance liquid chromatography
HF-LPME: hollow-fiber liquid phase microextraction
ICP-MS: inductively coupled plasma-mass spectrometry
ICP-MS: inductively coupled plasma–mass spectrometry
ICP-OES: inductively coupled plasma-optical emission spectrometry
IL: ionic liquid
LLE: liquid-liquid extraction
SDME: single-drop microextraction
SPE: solid phase extraction
USEPA: United States Environmental Protection Agency
VA-LPME: vortex-assisted liquid phase microextraction
WHO: World Health Organization
USA-DSL-MPME: ultrasound-assisted-dispersive solid-liquid multiple phase microextraction
HF-SPME: in-tube hollow fiber-solid phase microextraction
IP-RP-HPLC- ICP-MS: ion-pair reversed-phase high-performance liquid chromatography
DES-UALPME: deep eutectic solvent ultrasound-assisted liquid phase microextraction
